# MCL1 nuclear translocation induces chemoresistance in colorectal carcinoma

**DOI:** 10.1038/s41419-021-04334-y

**Published:** 2022-01-18

**Authors:** Dechen Fu, Luke Pfannenstiel, Abeba Demelash, Yee Peng Phoon, Cameron Mayell, Claudia Cabrera, Caini Liu, Junjie Zhao, Josephine Dermawan, Deepa Patil, Jennifer DeVecchio, Matthew Kalady, Andrew J. Souers, Darren C. Phillips, Xiaoxia Li, Brian Gastman

**Affiliations:** 1 Department of Inflammation and immunity, Lerner Research Institute, Cleveland Clinic, Cleveland, OH USA; 2 Cleveland State University, Cleveland, OH USA; 3 Case Western Reserve University, Cleveland, OH USA; 4 Pathology and Laboratory Medicine Institute, Cleveland Clinic, Cleveland, OH USA; 5 Digestive Health Research Institute, Case Western Reserve University, Cleveland, OH USA; 6 Center for Cancer Stem cell Research, Lerner Research Institute, Cleveland Clinic, Cleveland, OH USA; 7 Oncology-Discovery, AbbVie Inc, 1 North Waukegan Road, North Chicago, IL 60064 USA

**Keywords:** Targeted therapies, Senescence

## Abstract

Colorectal cancer (CRC) is one of the most common and deadliest forms of cancer. Myeloid Cell Leukemia 1 (MCL1), a pro-survival member of the Bcl-2 protein family is associated with chemo-resistance in CRC. The ability of MCL1 to inhibit apoptosis by binding to the BH3 domains of pro-apoptotic Bcl-2 family members is a well-studied means by which this protein confers resistance to multiple anti-cancer therapies. We found that specific DNA damaging chemotherapies promote nuclear MCL1 translocation in CRC models. In p53^null^ CRC, this process is associated with resistance to chemotherapeutic agents, the mechanism of which is distinct from the classical mitochondrial protection. We previously reported that MCL1 has a noncanonical chemoresistance capability, which requires a novel loop domain that is distinct from the BH3-binding domain associated with anti-apoptotic function. Herein we disclose that upon treatment with specific DNA-damaging chemotherapy, this loop domain binds directly to alpha-enolase which in turn binds to calmodulin; we further show these protein−protein interactions are critical in MCL1’s nuclear import and chemoresistance. We additionally observed that in chemotherapy-treated p53^−/−^ CRC models, MCL1 nuclear translocation confers sensitivity to Bcl-xL inhibitors, which has significant translational relevance given the co-expression of these proteins in CRC patient samples. Together these findings indicate that chemotherapy-induced MCL1 translocation represents a novel resistance mechanism in CRC, while also exposing an inherent and targetable Bcl-xL co-dependency in these cancers. The combination of chemotherapy and Bcl-xL inhibitors may thus represent a rational means of treating p53^−/−^ CRC via exploitation of this unique MCL1-based chemoresistance mechanism.

## Introduction

Colorectal carcinoma (CRC) accounts for almost a million deaths annually worldwide. The progressive accumulation of genetic alterations serve to inactivate tumor suppressor genes and activate oncogenes transforms neoplastic precursor lesion to a malignant state.

Numerous DNA damaging CRC therapies including doxorubicin and oxaliplatin, drive cell stress, inducing *p53*-mediated transcription of genes activating processes such as cell cycle arrest, senescence, and apoptosis. DNA damage induced apoptosis, occurs in part through *p53* response genes encoding pro-apoptotic Bcl-2 family proteins, which bind to and inhibit the anti-apoptotic Bcl-2 family members exemplified by Bcl-2, Bcl-xL, and MCL1. However, approximately 60% of human CRC harbor p53 mutations that suppress the effectiveness of these DNA damage-inducing therapies [1–4], highlighting a need to develop *p53*-independent treatment alternatives.

Many strategies aimed at improving interventions for cancer have focused on developing small molecular inhibitors capable of overcoming the ability of tumors to block apoptosis-inducing therapies [5–8]. The Bcl-2 selective inhibitor venetoclax is highly active alone or in the combination setting in patients with CLL [9, 10] or AML [11, 12], and efforts to understand its clinical activity in specific solid tumor indications are underway [13]. However, data derived in pre-clinical models of solid tumors emphasize a broader function for Bcl-xL and/or MCL1 in the maintenance of tumor cell survival [14–16]. Human CRC cell lines with *BCL2L2* amplifications have a propensity to depend upon Bcl-xL for survival [17]. Even though *MCL1* amplifications are low in CRC cell lines compared to other human tumor cell lines, MCL1 can still limit the activity of Bcl-xL inhibitors and other chemotherapeutic agents in CRC [17–19] and additional solid tumor indications [15, 20]. Although the role of MCL1 in regulating apoptosis is well established [21], emerging data highlight additional functions related to autophagy, mitochondrial respiration [22, 23], and DNA damage [24–26], which may also contribute to chemoresistance and cancer progression. Specifically, a unique loop domain within MCL1 regulates its phenotypic response to DNA damage [25]. We therefore sought to understand the mechanism of MCL1 in response to DNA damage, and potentially uncover strategies for exploiting this novel mechanism en route to improved treatment options for CRC.

Herein, we demonstrate that MCL1 during chemotherapy treatment localized mainly to the nucleus, which is facilitated by the formation of a novel tri-molecular complex with alpha-enolase (ENO1) and calmodulin (CaM), and that both of the latter two proteins are critical for this translocation. This is the first disclosure of the mechanism by which MCL1 moves from its classical mitochondrial compartment, and the data herein provides compelling evidence that the subcellular localization of MCL1 can modulate its function and impact processes beyond apoptosis [22, 23, 27]. Although the resulting accumulation of nuclear MCL1 drives resistance to DNA damaging agents, this chemoresistance can be overcome by Bcl-xL selective inhibitor A-1331852. Since we show via IHC that MCL1 and Bcl-xL are co-expressed at high levels in late-stage CRC patient samples, these data highlight a synthetic lethality between DNA damaging agents and Bcl-xL selective inhibitors that could be exploited for therapeutic benefit in this genomic defined and high-risk solid tumor population.

## Materials and methods

### Cell culture and drug treatments

The human colorectal cancer (CRC) cell lines SW480, HT29, and Colo205 cell lines and PDX cells sample were a gift from Dr. Matthew Kalady (Cleveland Clinic, OH) and was derived in his laboratory. HCT116 human colon cancer cell lines (p53^+/+^ and p53^−/−^) were provided by Dr. Bert Vogelstein (Johns Hopkins University, Baltimore, Maryland) as described before [25]. The p53 mutation status of CRC cell lines are HCT-116, wild type; HT29, p.R273H; Colo205, p.Y103fs*37 and p.Y103F; SW-480, double mutation p.R273H and p.P309S [28]. All cell lines were maintained in DMEM supplemented with penicillin/streptomycin, non-essential amino acids, and 10% FBS. HCT116 p53^−^^/−^ CRISPR *MCL1* and CRISPR *ENO1* cells are derivatives of HCT116 p53^−^^/−^, which stably express a transcript-specific CRISPR that knocks out endogenous *MCL1* and *ENO1* expression (Santa Cruz Biotechnology, Dallas, TX). All cell cultures were incubated at 37 °C in a humidified incubator containing 5% CO_2_. Cell viability was tested by using the CellTiter-Glo luminescent according to the manufacturer’s protocol (Promega, Madison, WI). Cells were tested after 2 to 3 days of treatment with drugs, and test results were normalized to cell without treatment. Cells were treated in fresh medium containing doxorubicin (100 ng/ml, Sigma, St Louis, MO) or oxaliplatin (10 μM) to induce cell death and CIS. Bcl-xL selective inhibitor A-1331852 (1 μM) and MCL1 selective inhibitor A-1210477 (10 μM) were provided by Abbvie Company, North Chicago. Cells were treated with Ophiobolin A (Oph, 5 µM, SML 1478, Sigma, St Louis, MO), and Calmidazolium chloride (CDZ 5 μM, 208665, Sigma, St Louis, MO).

### Plasmid transfections

To assess relevant amino acid residues in regulating the nuclear translocation activity of MCL1, we used PCR-based cloning to generate each of those deletions and cloning into pcDNA 3.1 vector. Flag-fused MCL1 was cloned into Flag-pcDNA 3.1 (Addgene #52535). The resulting plasmids were sequenced to ensure that they encoded the appropriate constructs. Transient plasmid transfection into the indicated cell lines was performed using Lipofectamine 2000 (Life Technologies, Carlsbad, CA) according to the instructions of the manufacturer. Briefly, 2 × 10^5^ cells/well in six-well plates or 1 × 105 cells/well in six-well plates on poly-l-lysine-coated glass coverslips were transiently transfected with 0.5 μg of WT *MCL1*, various *MCL1*-expressing constructs, or empty pcDNA3.1 vector (Invitrogen, Carlsbad, CA). The medium was changed after 24 h, and then cells were incubated for 48 h prior to verifying transgene expression by Western blot analysis. Flag-tagged MCL1 proteins were purified by using M2 affinity agarose gel (Sigma, St Louis, MO) according to the protocols from the manufacturer.

### Immunoblotting

Western blot analyses were performed as described previously [25]. The membranes were visualized using ECL reagents (GE Healthcare, Chicago, IL) or the Western Bright Quantum kit (Advansta, Menlo Park, CA). The primary antibodies used for Western blotting were anti-MCL1 (catalog no. D35A5, rabbit, dilution of 1:1000, Cell Signaling Technology, Danver, MA). Mouse anti-β-actin (Santa Cruz Biotechnology, Dallas, TX) at a dilution of 1:10,000 was used as a loading control. Anti-ENO1 antibodies were obtained from Abcam, Cambridge, UK (AB85086) and used at 1:1000 dilution. Anti-Flag antibodies were obtained from Sigma, St Louis, MO as described before.

### Mitochondria and nucleus fractionation

Mitochondria and Nucleus fractions were obtained using the Mitochondria Isolation Kit (Product No. 89874) and NE-PER™ Nuclear and Cytoplasmic Extraction kit (Product No.78833) for cultured cells and tumor samples from Thermo Fisher Scientific, Waltham, MA according to manufacturer’s instruction. Briefly, after 24–48 h doxorubicin (100 ng/ml) or oxaliplatin (10 μM) treatment, 2 × 10^7^ HCT116 p53^−/−^ cells, Colo205, SW480, HT29, CRISPR ENO1 cells or tumor cells were harvested by centrifuging at ~850 × g for 2 min, and mitochondria or Nucleus were isolated following the protocol provided by the kit. Mitochondria pellets and nucleus lysates were lysed in RIPA buffer. After removing the insoluble material by 14,000 × *g* centrifugation, protein from mitochondria or nucleus was quantified by BCA Assay kit (Product No. 23225, Thermo Fisher Scientific, Waltham, MA). For gel electrophoresis, 30 μg of total protein was loaded per lane and separated by SDS-PAGE, and then transferred to PVDF membrane (Bio-Rad, Hercules, CA). For immunoblot experiments, the membranes were sequentially blotted with, anti-Tom20 (Santa Cruz Biotechnology, Dallas, TX), anti-MCL1 (Cell Signaling Technology, Danver, MA) and Histone H3 (Santa Cruz Biotechnology, Dallas, TX) primary antibodies, and horseradish peroxidase-conjugated secondary antibody (Bio-Rad, Hercules, CA).

### GST fusion protein binding assay

GST-ENO1 constructs were obtained from ABM (PV349275) GST fusion proteins were produced in Dh5α *Escherichia coli* following the induction of expression by isopropyl 1-thio-β-D-galactopyranoside (Insight Biotechnology, Middlesex, UK) and purified using glutathione-Sepharose beads (Amersham Biosciences, Little Chalfont, UK). GST pull-down assay was finished according to manufacturer’s instruction The purified MCL1 proteins were incubated with GST or GST-ENO1 fusion proteins bound to glutathione-Sepharose beads in bead-binding buffer (50 mM potassium phosphate, pH 7.5, 150 mM KCl, 1 mM MgCl_2_, 10% glycerol, 1% Triton X-100) and protease inhibitors mixture from Roche Applied Science, Penzberg, Germany. The mixtures were incubated at 4 °C for 2 h with rotation. The beads were then pelleted and washed five times in ice-cold bead-binding buffer. Finally, beads were re-suspended in SDS sample buffer, and the proteins were resolved on SDS-polyacrylamide gels and subject to western blotting.

### Immunoprecipitation

For each immunoprecipitation experiment, HCT116 p53^−/−^ cells with CRISPR *MCL1* were transfected with Flag-tagged *MCL1* or *MCL1* mutants in pcDNA3.1 or empty vector. Immunoprecipitation was essentially carried out as described [29]. Briefly, 48 h after transfection, cells were harvested and re-suspended in ice-cold lysis buffer containing 20 mM Tris/HCl (pH 7.4), 150 mM NaCl, 2 mM EDTA, 10% glycerol, 1% Triton X-100, and protease inhibitor mixture (Roche Applied Science). Lysates were precleared and then incubated with either M2 anti-flag monoclonal antibody (Sigma, St Louis, MO) or rabbit anti-MCL1 polyclonal antibody (Cell Signaling Technology, Danver, MA) at 4 °C for 1 h, and protein A-Sepharose beads (Pharmacia, New Jersey) were added to pull down the immunocomplexes. The beads were washed five times in washing buffer containing 0.2% Triton X-100, 20 mM Tris/HCl (pH 7.4), 150 mM NaCl, 2 mM EDTA,10% glycerol before being re-suspended in SDS sample buffer and subjected to SDS-PAGE. Immunoblotting was performed using, where appropriate, anti-ENO1 antibody (Cell Signaling Technology, Danver, MA), anti-CaM antibody (Santa Cruz Biotechnology, Dallas, TX).

### Immunofluorescence staining

Cultured cells were treated with doxorubicin or oxaliplatin for 2−3 days to induce senescence. Tissue sections were obtained from animal treated with drugs or no-treatment controls. Immunofluorescence was performed as described previously [25]. Anti-PML (catalog no. sc-966, 1:100, Santa Cruz Biotechnology, Dallas, TX), anti-γH2Ax (ser-139, 1:100, BioLegend, San Diego, CA) and anti- Cleaved Caspase 3 (D175, 1:100, Cell Signaling Technology, Danver, MA). Cells were incubated with anti-mouse or anti-rabbit secondary conjugated with Alexa Fluor (1:500, BioLegend, San Diego, CA). Ten representative fields were selected randomly for the quantification of PML and γH2AX nuclear body formation. The number of foci present in each cell nucleus was counted manually in 30 transfected and drug-treated cells as well as in 30 transfected but not drug-treated cells using a Leica DM5500 B fluorescence microscope at ×40 oil immersion (Leica, Wetzlar, Germany).

### Senescence β-Galactosidase assays

Cultured cells were assayed for senescence-associated (SA) β-gal expression as described previously [25]. Briefly, cells were washed and fixed with 2% paraformaldehyde (Thermo Fisher Scientific, Waltham, MA) for 5 min at room temperature. Cells were then incubated in the dark for up to 16 h in a staining solution containing 1 mg/ml X-gal (Gold Biotechnology, St. Louis, MO) in dimethylformamide (Acros Organics, Fair Lawn, NJ), 40 mm of a 0.2 m citric acid/sodium phosphate buffer (pH 6.0), 5 mm potassium ferrocyanide (Sigma, St Louis, MO), 5 mm potassium ferricyanide (Sigma, St Louis, MO), 150 mm sodium chloride, and 2 mm magnesium chloride. Stained cells were then visualized under an inverted bright-field microscope. Ten representative fields were selected randomly for the quantification of β-gal-positive cells as a percentage of the total cell number. For tissue analysis, fresh-frozen tissue samples were cut into 8 μm sections, fixed with 1% paraformaldehyde for 1 min, and washed with PBS, followed by overnight incubation with SA β-gal staining solution.

### Inflammation CRC mouse model

All animal experiments were confirmed to our animal protocols that were reviewed and approved by the Institutional Animal Care and Use Committee. B6 genetic background mice were obtained from the Jackson Laboratory. For azoxymethane/dextran sodium sulfate treatment experiments, male mice for each genotype were given a single intraperitoneal administration of AOM (10 mg/kg body weight). Seven days later, these mice were randomly divided into two groups fed with 1.25% DSS in drinking water for four cycles. At the end of the experiments, some of the colons from each group were fixed for counting tumors, and histologic staining and immunofluorescence staining were as described previously.

### In vivo experiments

We injected HCT116 p53^−/−^ control and CRISPR ENO1 cells subcutaneously into NSG mice to establish xenograft tumors [25]. Typically, this takes ~10 days before a palpable tumor develops that is around 4 mm in diameter. We then treat these mice with either doxorubicin (1.2 mg/kg i.p. q3d) or A-1331852 (25 mg/kg PO via oral gavage q1d), an experimental Bcl-xL inhibitor A-1331852 provided through a partnership with AbbVie, Inc., or the combination of the two drugs. At the conclusion of the experiment, tumor tissue will then be harvested and analyzed for gene expression and markers of cell death and senescence. In the dual-treatment group, drugs will be administered 4 h apart. Treatment groups: Tumor alone, Tumor + 1.2 mg/kg doxorubicin, Tumor + 25 mg/kg A-1331852 and Tumor + 25 mg/kg A-1331852 + 1.2 mg/kg doxorubicin. We used 5 mice per group and 40 mice total without randomization.

### PDX mouse model

The research protocol was approved by Cleveland Clinic and all patients provided appropriate informed consent. Human tumor tissue samples were obtained from cancer patients. Freshly resected human tumor samples were transplanted subcutaneously (SC) into NSG mouse. SC PDX tumor were treated with doxorubicin (1.2 mg/kg i.p. q3d) for 14 days. The mouse was euthanized and PDX tumor were collected and fixed in OCT with liquid nitrogen. Frozen sections (8um were stained with hemotoxylin and eosin (H&E) for initial histopathological evaluation. For immunohistochemical (IHC) staining, we used antibodies against the MCL1, γH2Ax, and cleaved Caspase 3 as described above. IHC staining of MCL1 was visualized with Dako Envision + /HRP kit according to the manufacturer’s recommended procedure.

### Human tissue specimens

Following IRB approval, primary CRC and metastatic liver tissue samples were collected. The FFPE tissue samples were deparafinized then rehydrated through graded ethanol solutions. Bcl-2 samples underwent antigen retrieval by incubating in Citrate Buffer pH 6 for 20 min at 60 °C. Dako EnVision System-HRP (DAB) staining kits specific for both rabbit and mouse were used appropriately. Endogenous peroxidase was blocked by incubating in Peroxidase Block solution (Dako EnVision System-HRP) for 10 min. Tissues were blocked with 1% BSA/PBS solution for 15 min. Primary antibodies specific to MCL1 (rabbit, Abcam,1:100), Bcl-xL (rabbit, 1:100, Cell Signaling Technology, Danver, MA), and Bcl-2 (mouse, LifeSpan, pre-diluted) were incubated at room temperature for 1 h. Appropriate IgG antibodies (rabbit & mouse, 1:200, Santa Cruz Biotechnology, Dallas, TX) were used on one of the tissue samples as a negative control. Labeled Polymer-HRP (rabbit & mouse, Dako EnVisionSystem-HRP) were incubated for 30 min on the appropriate tissues. DAB + Substrate Buffer (Dako EnVision System-HRP) activated with DAB + Chromogen (Dako EnVision System-HRP) was incubated for 10 min. Samples were counter-stained with 1:1 hematoxylin (Dako, Agilent, Santa Clara,, CA) then dipped in PBS. Samples were then mounted with xylene based mounting medium.

Variables collected include: tumor primary site, survival time, vital status, MCL1, Bcl-xL, and Bcl-2 staining status. Staining was categorized as follows: Negative/low or medium/high by generating a composite score based on intensity (weak, moderate, strong) and % positive tumor cells (<30%, 30–70%, >70%). Nuclear and/or cytoplasmic MCL1 and cytoplasmic Bcl-xL and Bcl-2 staining were considered positive. This data was collected by the authors, with prior approval from the Cleveland Clinic Institutional Review Board.

Odds ratios (OR) were calculated using a two-by-two frequency table between Bcl-xL and MCL1. Chi-square tests for independence were used to evaluate the relationship between two categorical variables. Statistical significance was achieved with a value less than 0.05. The data analysis was performed using R statistical software (version 3.4.5, R Foundation for Statistical Computing, Vienna, Austria).

## Results

### Chemotherapy causes MCL1 nuclear translocation in CRC cell lines in response to DNA damage

**Fig. 1:**
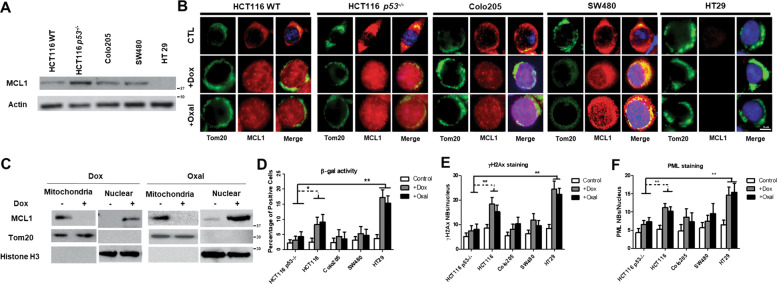
MCL1 protein nuclear translocation can be induced by chemotherapy treatment. **A** Western blot for MCL1 protein level in CRC cell lines HCT116 wild type (WT), HCT116 p53^−/−^, Colo205, SW480, and HT29. Actin was used as loading controls. Data are representative of three independent experiments. **B** Representation of confocal microscopy images of CRC cell lines: HCT116 WT, HCT116 p53^−/−^ Colo205, SW480, and HT29 treated with doxorubicin (100 ng/ml) (middle panel) and oxaliplatin (10 μM) and no drug treatment control (top panel). Cells were stained with anti-Tom20 (Green) and anti-MCL1 (Red) antibodies. Nucleus were stained with DAPI (Blue). **C** Mitochondria extraction and nuclear extraction were isolated from HCT116 p53^−/−^ cells treated with doxorubicin (100 ng/ml) (left panel) or oxaliplatin (10 μM) (right panel). No drug treatment was used as control. Western blot was used to detect the protein level of MCL1. Tom 20 was used as an indicator of mitochondria and Histone H3 was used as an indicator of the nucleus. Data are representative of two independent experiments. Quantitative analysis of chemotherapy-induced senescence (CIS) in CRC cell lines and analyzed for β-gal activities (**D**), γH2AX nuclear bodies formation (**E**), and PML nuclear bodies formation (**F**). T-test is used to calculate the *p*-value, *, *p* < 0.05 and **, *p* < 0.01 for the indicated doxorubicin or oxaliplatin treated cell line compared with HCT116 p53^−/−^ cells. Error bars represent +/−S.D.

While the classical pro-survival activities of MCL1 occur at the mitochondria via its BH3 domain, we previously reported that MCL1 can also promote chemoresistance through an alternative mechanism [25, 30, 31]. To further understand this mechanism, we first assembled a small panel of CRC cell lines that possess differing levels of MCL1 (Fig. 1A) and treated these cell lines with either doxorubicin or oxaliplatin. By immunostaining, both doxorubicin and oxaliplatin stimulate the trafficking of MCL1 from mitochondria to the nucleus (Figs. 1B and S1A). The nuclear translocation of MCL1 protein in HCT116 p53^−/−^ cells was additionally confirmed by mitochondria and nuclear extraction (Fig. 1C). Similar nuclear translocation of MCL1 was observed in Colo205, SW480, and HT29 when treated with doxorubicin and oxaliplatin, although partial translocation was found in SW480 (Fig. S1B). We and others’ have previously established that doxorubicin induces DNA damage in CRC cell lines and reduces their cell viability in vitro. We show that p53^−/−^ HCT116 cells exhibited both MCL1 nuclear translocation and resistance to chemotherapy-induced β-galactosidase activity (senescence marker), formation of nuclear γH2Ax (senescence and aging marker) and PML foci (DNA damage marker, Fig. 1D–F), compared to p53^WT^ HCT116 or HT29 cells possessing low MCL1 expression. Double p53-mutant SW480 (R273H, P309S), HT29 (R273H) and Colo205 (Y103fs*37, Y103F) have loss of p53 function [28]. It is not surprising that SW480 and Colo205 cells, which are considered functionally p53-deficient, are resistance to chemotherapy-induced senescence when MCL1 translocate into the nucleus, although statistically insignificant compared to p53^WT^ HCT116 cells. These data indicate that the MCL1 nuclear translocation is associated with MCL1-mediated chemoresistance in cancer cells lacking functional p53.

### MCL1 nucleus translocation protects cells against senescence in the absence of p53

**Fig. 2:**
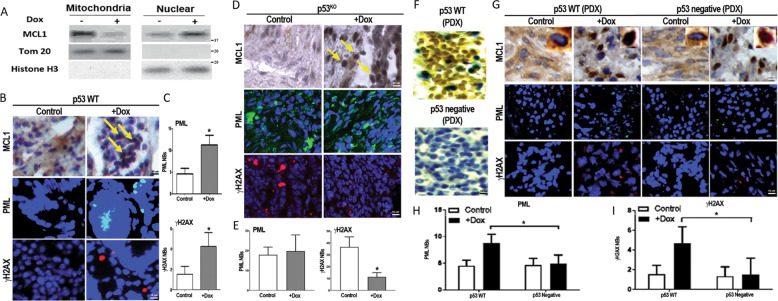
Chemotherapy treatment induces MCL1 protein nuclear translocation in Inflammatory CRC mouse model and PDX. **A** Mitochondria extraction and nuclear extraction were isolated from CRC mouse model treated with doxorubicin 1.2 mg/kg. No drug treatment was used as control. Western blot was used to detect the protein level of MCL1. Tom 20 was used as an indicator of mitochondria and Histone H3 was used as an indicator of the nucleus. Data are representative of two independent experiments. **B** Representation of microscopy images of inflammatory CRC mouse model. Tissues were stained with MCL1, PML, and γH2AX nuclear bodies. Yellow arrows show the nucleus located MCL1 proteins. **C** Quantitative analysis of chemotherapy-induced senescence (CIS) in CRC mouse model and analyzed for PML and γH2AX nuclear bodies. *p < 0.05 for the indicated doxorubicin treated tumor compared with no drug treatment controls. Error bars represent +/−S.D. **D** Representation of immunofluorescence microscopy images of p53^KO^ tumors with or without doxorubicin treatment. Tissues were stained with MCL1, PML, and γH2AX nuclear bodies. **E** Quantitative analysis of CIS in p53^KO^ model for PML and γH2AX nuclear bodies. T-test was performed to calculate *p* values, *, *p* < 0.05. **F** Representation of microscopy images of PDX model with wild type and p53 negative. The tissue was stained with anti-p53 antibodies. **G** Representation of microscopy images of PDX models. After injected with CRC cells, mice received 1.2 mg/kg doxorubicin every 3 days or were left untreated. Tissues were stained with MCL1, PML, and γH2AX nuclear bodies. The staining of MCL1 protein in single cells was shown in an enlarged picture on the top of the right side within each represented data. **H, I** Quantitative analysis of CIS in PDX model and analyzed for, PML and γH2AX nuclear bodies. Error bar represent +/−S.D. T-test was used to calculate the *p* values, *, *p* < 0.05 for the indicated doxorubicin treated PDX with and without wild type p53.

We next determined the MCL1 nuclear translocation in vivo by employing an inducible colon carcinogenesis mouse model, using dextran sodium sulfate (DSS). We started with a pilot experiment treating p53^WT^ C57BL/6 immune-competent mice to undergo colon tumorigenesis with doxorubicin. Subcellular fractionation confirmed that doxorubicin-induced MCL11 nuclear translocation in vivo (Fig. 2A). To further interrogate the role of p53 in MCL1-mediated chemoresistance in vivo, we utilized p53^WT^ and p53^KO^ mice and induced colon cancer using DSS. In the presence of doxorubicin, MCL1 translocated into the nucleus in both p53^WT^ and p53^KO^ mice (Fig. 2B, D). In p53^WT^ animals, MCL1 nuclear translocation triggered upregulation of chemotherapy-induced senescence-associated DNA damage markers γH2Ax and PML foci in the transformed cells (Fig. 2B, C). Contrarily, doxorubicin-treated p53^KO^ mice exhibited γH2Ax downregulation, but no statistical change in PML expression (Fig. 2D, E).

We also investigated whether MCL1 nuclear translocation correlated with chemoresistance in vivo using patient-derived xenografts (PDX). *W*e examined PDXs from colorectal cancer patients obtained from our institution, with known differential expression of p53 (Fig. 2F). Consistent with our observations in the cell lines and DSS-induced colon cancer animal model above, doxorubicin treatment caused MCL1 to translocate into the nucleus in p53^WT^ and p53^negative^ PDXs (Fig. 2G). Consistently p53^negative^ status prevented upregulation γH2Ax and PML foci expression (Fig. 2H, I). These data support the role of nuclear MCL1 in chemoresistance in p53-deficient colorectal cancer in vitro and in vivo.

### Chemotherapy-induced MCL1 nuclear translocation via unique loop domain

**Fig. 3:**
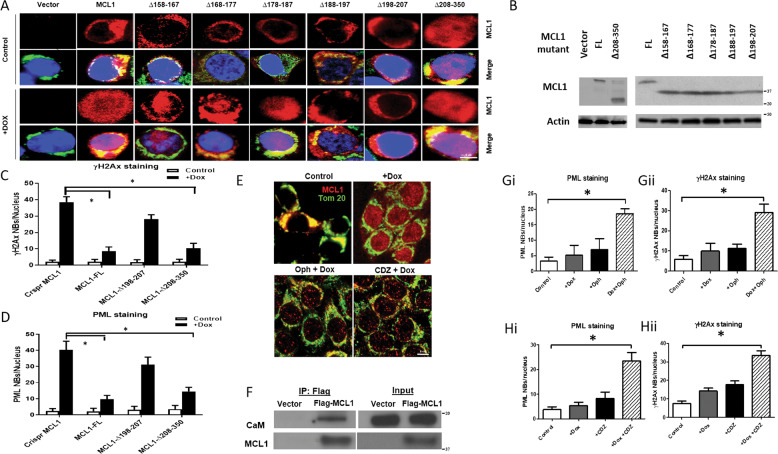
The loop domain of MCL1 affects its nuclear translocation of MCL1 is affected by its loop domain and calmodulin. **A** Representation of confocal microscopy images of HCT116 p53^−/−^ with CRISPR *MCL1* cells. Cells were transiently transfected with empty vector, wild type *MCL1*, mutant *MCL1* with ten amino acids deletion within the loop domain, and *MCL1* mutant with C-terminal deletion. Doxorubicin (100 ng/ml) (lower panel) treatment was used to induce MCL1 nuclear translocation and no drug treatment was used as control (top panel). Cells were stained with anti-Tom20 (Green) and anti-MCL1 (Red) antibodies. Nucleus was stained with DAPI (Blue). **B** Western blot for wild type and mutant MCL1 protein level in HCT116 p53^−/−^ with CRISPR *MCL1*. Actin was used as loading controls. Data are representative of two independent experiments. Quantitative analysis of chemotherapy-induced senescence (CIS) in CRISPR *MCL1* HCT116 p53^−/−^ cells with expression of wild type and mutant *MCL1* and analyzed for γH2AX nuclear bodies formation (**C**), PML nuclear bodies formation (**D**). T-test was used to calculate the *p* values. *, *p* < 0.05 for the indicated *MCL1* expression cells treated with doxorubicin compared with vector control cells. Error bars represent +/−S.D. **E** HCT116 p53^−/−^ cells were treated with the CaM inhibitor ophiobolin A (Oph, 5 µM) or Calmidazolium chloride (CDZ 5uM) for two hours followed by treatment with 100 nM doxorubicin for 24 h. Cells were then fixed and stained for MCL1 (red) and Tom20 (green). No drug treatments were used as control. **F**. Co-immunopreciptation assay using anti-Flag antibody to pulldown CaM in MCL1 knockout HCT116 p53^−/−^ cells with transient transfected with Flag-tagged MCL1 or empty vector. Input is 10% of the material used for immunoprecipitation. **G**, **H** Quantitative analysis of chemotherapy-induced senescence (CIS) in HCT116 p53^−/−^ cells treated with doxorubicin and/or CaM inhibitor Oph (G) or CDZ (H) and analyzed for PML nuclear bodies formation (Gi and Hi) or γH2AX nuclear bodies formation (Gii and Hii). T-test was used to calculate the *p* values. *, *p* < 0.05 for the indicated HCT116 p53^−/−^ cells treated with drugs compared with vector control cells. Error bars represent +/−S.D.

We previously reported that MCL1-mediated chemoresistance requires a novel loop domain containing residues 194–207 [25]. To confirm that MCL1 nuclear translocation was required for chemoresistance while further elucidating which protein domain was engaged in this process, we expressed the previously described truncated variants of MCL1 [25] in p53^−/−^ HCT116 cells deficient in endogenous MCL1, and tested the impact of doxorubicin on their nuclear localization. Interestingly, deletion mutants (∆198–207) within the loop domain but not the BH3 domain (∆208–350) nor residues near the loop domain (∆158–167, ∆168–177, ∆178–187) impaired chemotherapy-induced nuclear translocation of MCL1 (Fig. 3A, B). The deletion mutants (MCL1-∆198–207) within the loop domain showed substantially lesser suppression of DNA damage markers, indicating lower chemoresistance (Fig. 3C, D). These data suggest that not only is the chemoresistance of MCL1 dependent on its ability to translocate to the nucleus but that the latter process requires the loop domain.

### MCL1 nuclear translocation is sensitive to calmodulin inhibition

MCL1 does not have a canonical nuclear localization sequence (NLS) [29], an observation supported by the additional finding that the importin inhibitor ivermectin (which inhibits NLS-mediated nuclear import) failed to suppress MCL1 nuclear translocation (Fig. S1C); both findings collectively suggesting that MCL1 nuclear translocation relies on an importin-independent pathway. To understand the mechanism by which MCL1 traffics to the nucleus, we first developed an assay designed to identify any proteins that interact with MCL1 loop domain that might be required for both MCL1 nuclear translocation and MCL1 ability to mediate chemoresistance. We employed a MCL1-Δ208–350 mutant as bait (no BH domains to mediate protein-protein interactions) and performed mass spectrometry analysis on the complexes that were immunoprecipitated with anti-MCL1 antibodies. Since the proline residue at bp198 within the loop domain is required for MCL1 to mediate chemoresistance [25], mutant MCL1-Δ208–350-P198A was used as a control. This experiment identified some candidate proteins that, in a doxorubicin-inducible manner, interacted with MCL1-Δ208–350, but not MCL1-Δ208–350-P198A (Fig. S2A, B).

Among these interacting candidates, CaM was previously shown to mediate the nuclear transport of cytosolic proteins that have no clear NLS [32]. Using two different CaM inhibitors, Ophiobolin A or calmidazolium chloride, we found that CaM activity was required for doxorubicin-induced MCL1 nuclear translocation (Fig. 3E). We validated CaM/MCL1 binding by co-immunoprecipitation (Fig. 3F). Treatment with CaM inhibitors Ophiobolin A (Fig. 3G) or calmidazolium chloride (Fig. 3H) increased the number of nuclear PML (Fig. 3Gi, Hi) and γH2Ax foci (Fig. 3Gii, Hii). These results suggest that CaM-mediated MCL1 nuclear translocation may be required for MCL1-mediated chemoresistance.

### ENO1 mediates calmodulin-dependent MCL1 nuclear translocation

**Fig. 4:**
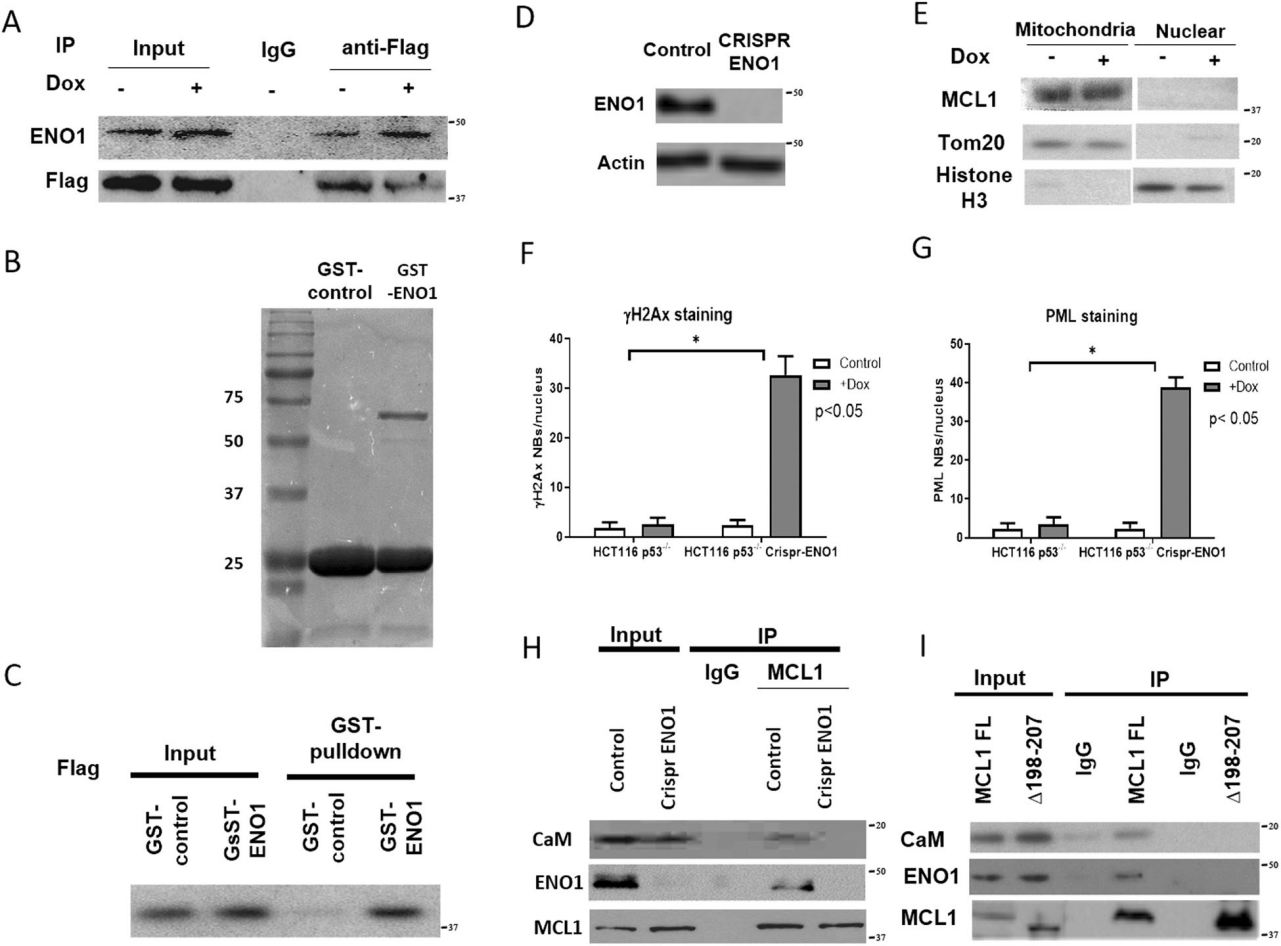
ENO1 interacts with MCL1 and promote its nuclear translocation under CIS condition. **A** Co-immunopreciptation assay using anti-Flag antibody to confirm ENO1 interact with MCL1 under CIS condition in *MCL1* CRISPR HCT116 p53^−/−^ cells with transient transfected with Flag-tagged MCL1. Input is 10% of the material used for immunoprecipitation. **B** Migration of GST tagged ENO1 protein is detect by Coomassie Blue staining. **C** GST pulldown assay using GST tagged ENO1 as bait to precipitate MCL1 protein, GST empty vector was used as control. **D** Western blot for ENO1 protein level in HCT116 p53^−/−^ with CRISPR *ENO1* and control cells. Actin was used as loading controls. Data are representative of two independent experiments. **E**. Mitochondria extraction and nuclear extraction were isolated from HCT116 p53^−/−^ CRISPR *ENO1* cells treated with doxorubicin (100 ng/ml). No drug treatment was used as control. Western blot was used to detect MCL1 protein level. Tom 20 was used as an indicator of mitochondria and Histone H3 was used as an indicator of the nucleus. Data are representative of two independent experiments. Quantitative analysis of chemotherapy-induced senescence (CIS) in HCT116 p53^−/−^ with CRISPR *ENO1* and control cells and analyzed for γH2AX nuclear bodies formation (**F**) or PML nuclear bodies formation (**G**). T-test is used to calculate the *p-*value. *, *p* < 0.05 for the indicated doxorubicin treated cells compared with no drug treatment controls. Error bars represent +/−S.D. **H** Co-immunopreciptation assay using anti-MCL1 antibody to pull down CaM (Cam) (top panel) and ENO1 (Middle panel) in HCT116 p53^−/−^ with CRISPR *ENO1* cells. Input is 10% of the material used for immunoprecipitation. **I** Co-immunopreciptation assay using anti-MCL1 antibody to pull down the CaM (Top panel) and ENO1 (Middle panel) in *MCL1* CRISPR HCT116 p53^−/−^ cells with transient transfected with wild type or mutant *MCL1*. Input is 10% of the material used for immunoprecipitation.

Intriguingly, among our list of candidate proteins that interact with MCL1 *via* the loop domain were interleukin enhancer-binding factor 2 (ILF2) and ENO1, both of which carry a CaM-binding motif [33]. This led us to hypothesize that MCL1 and CaM may be in a complex with ILF2 and/or ENO1. We confirmed the MCL1/ILF2 and MCL1/ENO1 interactions using co-immunoprecipitation (Fig.4A, data not shown). However, since ILF2-deficiency did not affect doxorubicin-induced MCL1 nuclear translocation, and since recombinant ILF2 failed to pull down MCL1 in vitro (data not shown), we hypothesized that only the MCL1/ENO1 interaction is likely to be biologically relevant.

The enzyme ENO1 acts in the glycolytic pathway and can translocate into the nucleus under certain conditions [34]. Here, we demonstrated a MCL1/ENO1 interaction by using recombinant ENO1 to pull down purified MCL1 in vitro (Fig. 4B, C). Furthermore, ENO1 deficiency blocked doxorubicin-induced MCL1 nuclear translocation (Figs. 4D, E and S1D) and rendered the otherwise resistant p53^−/−^ HCT116 cell line sensitive to doxorubicin-induced DNA damage (Fig. 4F, G). MCL1 and CaM also failed to co-immunoprecipitate in ENO1-deficient cells (Fig. 4H). Co-immunoprecipitation studies also confirmed that only full-length MCL1, but not a loop domain deletion mutant of MCL1, co-immunoprecipitated with both CaM and ENO1 (Fig. 4I). This suggests ENO1 may serve as a bridge between MCL1 and CaM *via* its CaM binding motif, and that MCL1, CaM, and ENO1 may be in a MCL1 loop-domain-dependent, tri-molecular complex. Together, the data indicate that ENO1 mediates CaM-dependent MCL1 nuclear translocation, which is requisite for MCL1 to promote chemoresistance, as illustrated in our proposed model (Fig. S3A).

### MCL1 and Bcl-xL co-expression in primary CRC patient

**Fig. 5:**
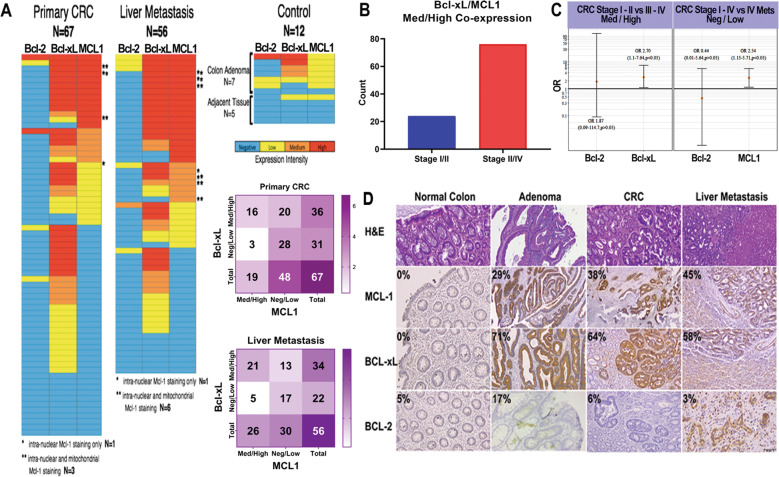
Co-localization of overexpression MCL1 with Bcl-xL in clinical CRC samples. **A** Representation of protein expression for primary CRC and liver metastasis tissues. Rows represent individual patients. Primary CRC includes stages 1, 2, 3, and 4. Colon adenomas and adjacent tissues were used as control tissues. Expression patterns (negative, low, medium, and high) are shown for Bcl-2, Bcl-xL, and MCL1. Intra-nuclear MCL1 expression is marked with a single asterisk. Both nuclear and cytoplasmic expressions are marked with two asterisks. Two by two tables represent Bcl-xL vs MCL1 co-overexpression. Staining was categorized as follows: negative/low or medium/high by generating a composite score based on intensity (weak, moderate, and strong) and % positive tumor cells (<30%, 30–70%, >70%). Nuclear and/or cytoplasmic MCL1 and cytoplasmic Bcl-xL and Bcl-2 staining were considered positive. Chi-square tests were used to correlate marker expression with tumor location, stage, and grade. **B** Bar plot representing percentages of Bcl-xL/MCL1 Med/High co-expression, 95% CI (0.32–0.71, *p* < 0.05) in only CRC samples. **C** On the left: Forest plot representing OR for Bcl-2 Med/High and Bcl-xL Med/High between stage I/II and III/IV. On the right: Forest plot representing OR for Bcl-2 Neg/Low and MCL1 Neg/Low between Stage I−IV and IV Liver Mets. The graph is presented in a log scale. **D** Representative of primary CRC and mCRC tissues stained for various genes using 3,3’-Diaminobenzidine (DAB) Chromogen (Dako). The intensity of brown color is proportional to increased gene expression and graded: negative, low, medium, and high. Adenomas and normal adjacent tissues were used as positive and negative controls, respectively. Percentages represent only medium/high MCL1, Bcl-xL, and Bcl-2 expression within the sample.

Small molecule inhibitors that selectively target individual members of the Bcl-2 family of anti-apoptotic proteins [5, 7, 8] are being evaluated in patients with hematologic and solid tumors. However, a comprehensive understanding of how the pro-survival Bcl-2 family members expression in CRC patients influences the clinical development of small molecule inhibitors targeting these proteins remains largely unexplored, particularly in the combination setting. To delineate the potential of MCL1 to limit the clinical activity of chemotherapy such as doxorubicin or oxaliplatin, we evaluated the expression of MCL1 and the related family members Bcl-2 and Bcl-xL in a large panel of CRC tumor biopsies (*n* = 133) by IHC. In primary CRC, Bcl-2 expression was low/negative in 65 of 67 (97%) samples (*p* < 0.05). Medium/high Bcl-xL expression was found in 36 of 67 (54%) primary CRCs (*p* < 0.05). 19 of 67 (28%) primary CRCs (*p* < 0.05) had medium/high MCL1 expression, of which 1 of 67 (1%) showed intra-nuclear staining and 3 of 67 (4%) showed both mitochondrial and intra-nuclear staining (Fig. 5A). In liver metastases, Bcl-2, Bcl-xL and MCL1 expression was low/negative in 55 of 56 (98%, *p* < 0.05), medium/high in 33 of 56 (60%, *p* < 0.05) and medium/high in 26 of 56 (46%, *p* < 0.05) samples, respectively. Within the MCL1-positive samples, 1 of 56 (2%) showed intra-nuclear staining and 6 of 56 (11%) showed both mitochondrial and intra-nuclear staining (Fig. 5A). It should be noted that, in these set of samples, we observed a heretofore unexplored pattern of Bcl-xL and MCL1 co-expression with medium/high MCL1 expression correlated positively with Bcl-xL but not with Bcl-2 expression (Fig.5B). For both primary CRC and the liver metastases, the probability of a sample being in the MCL1 medium/high group is higher for a sample in the Bcl-xL medium/high group when compared to a sample in the Bcl-xL low/negative group (Fig. 5B). This co-expression of Bcl-xL and MCL1 was more common in late stages III−IV of CRC compared to early stages I−II (Fig. 5C). Representative staining samples were illustrated in Fig. 5D. Our data emphasize the co-operative role that MCL1 and Bcl-xL expression play in the maintenance and progression of human CRC.

### Nuclear translocation of MCL1 creates vulnerability to Bcl-xL inhibitors

**Fig. 6:**
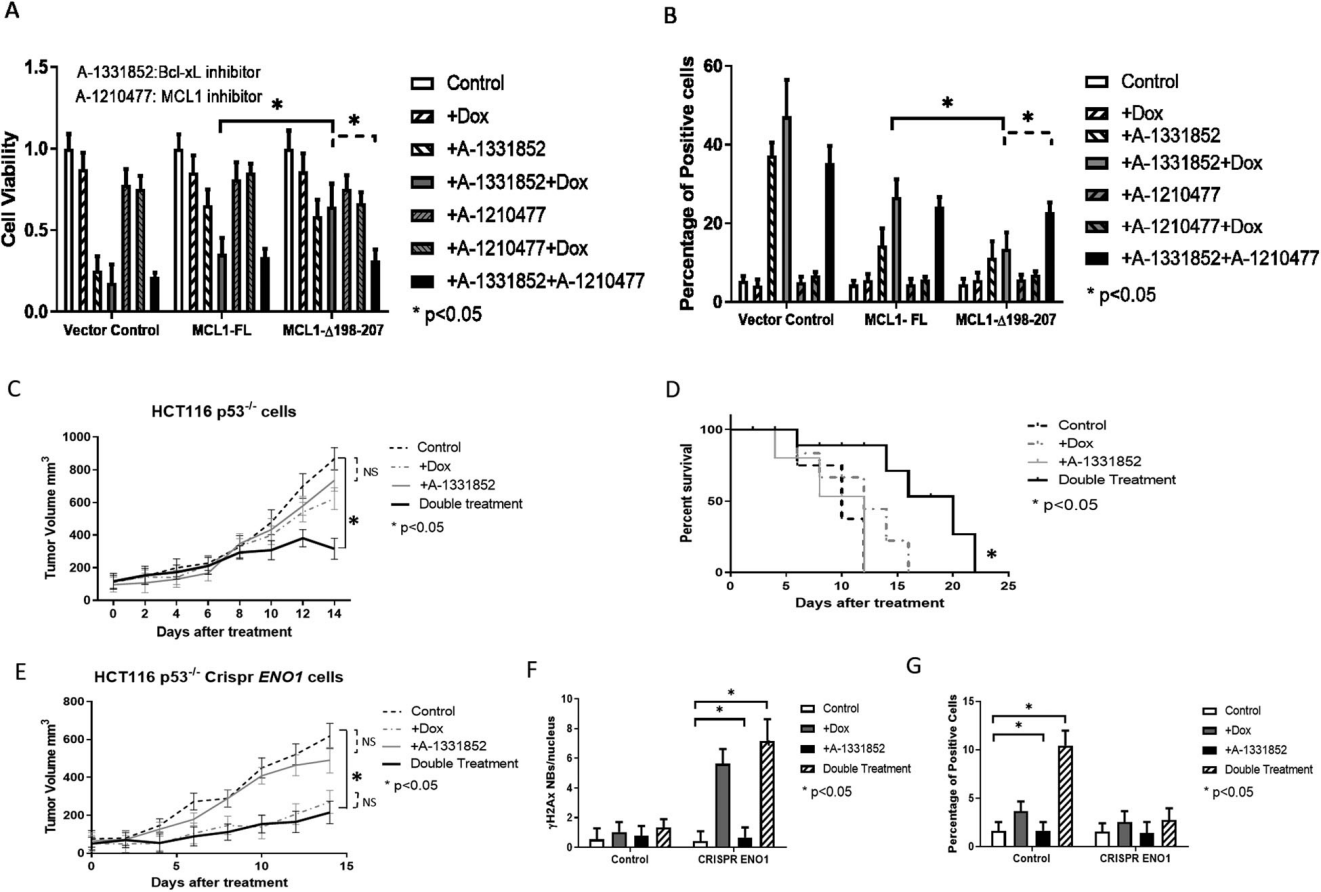
Nuclear translocation of MCL1 proteins affect its apoptosis activity in vitro and in vivo. **A**. Quantitative analysis of cell viability test in HCT116 p53^−/−^ with CRISPR *MCL1* cells treated with doxorubicin (100 ng/ml), Bcl-xL inhibitor A-1331852 (1uM), MCL1 inhibitor A-1210477 (10uM), or combined treatments for the indicated HCT116 p53^−/−^ with CRISPR *MCL1* cells expressing wild type or mutant *MCL1*. The solid line indicates the comparison of cells expressing full length or mutant *MCL1* treated with Bcl-xL inhibitor A-1331852 with doxorubicin. The dotted line indicates the comparison of cells expressing mutant *MCL1* treated with Bcl-xL inhibitor A-1331852 with doxorubicin or MCL1 inhibitor. T-test is used to calculate the *p-*value. *, *p* < 0.05. Error bars represent +/−S.D. **B** Quantitative analysis of cleaved caspase 3 staining in HCT116 p53^−/−^ with CRISPR *MCL1* cells expressing WT or mutant *MCL1*. Cells were treated with doxorubicin, Bcl-xL inhibitor A-1331852, MCL1 inhibitor A-1210477, or combined treatments. T-test is used to calculate the *p*-value, *, *p* < 0.05. Error bars represent +/−S.D. The solid line and dotted line are explained above. **C** tumor growth curves of HCT116 p53^−/−^ xenografts. Mice (*N* = 5 for each group) were injected with HCT116 p53^−/−^ cells. Mice received either doxorubicin (1.2 mg/kg i.p. q3d) or A-1331852 (25 mg/kg PO via oral gavage q1d), no drug treatment group were used as control. Error bars represent +/−S.D. T-test is used to calculate the *p*-value. *, *p* < 0.05 for tumor growth in the double treatment group, comparing with no drug treatment control group. N.S. indicate no significance according to *p-*value. **D**. mice (*N* = 5 for each group) survival data, with injected with doxorubicin and/or A-1331852 as indicated above. T-test is used to calculate the *p* value and *, *p* < 0.05 for the indicated doxorubicin and double treatment treated tumors (black solid line) comparing with no drug treatment controls (black dash line). N.S. indicate no significance according to *p*-value. Mice death is calculated as either mice that died during treatment or when tumor size reached 15 mm in diameter. **E** Tumor growth curves of xenograft HCT116 p53^−/−^ cells with CRISPR *ENO1*. Mice (*N* = 5 for each group) were injected with HCT116 p53^−/−^ cells with CRISPR *ENO1*. Mice received either doxorubicin and/or A-1331852 as indicated above. No drug treatment group was used as control. Error bars represent +/−S.D. T-test is used to calculate the *p*-value. *, *p* < 0.05 for tumor growth in doxorubicin and double treatment group, comparing with no drug treatment control group. N.S. indicate no significance according to *p*-value. **F** Quantitative analysis of chemotherapy-induced senescence (CIS) by staining γH2AX nuclear bodies for xenograft tumor tissue sections from (**A** and **B**). T-test is used to calculate the *p-*value. *, *p* < 0.05 for the indicated doxorubicin and double treatment treated tumors comparing with no drug treatment controls. Error bars represent +/−S.D. **G** Quantitative analysis of cell death by staining with cleaved caspase 3 for xenograft tumor tissue sections from (**C** and **D**). T-test is used to calculate the *p*-value. *, *p* < 0.05 for the indicated doxorubicin and double treatment treated tumors comparing with no drug treatment controls. Error bars represent +/−S.D.

It is well established that the anti-apoptotic function of MCL1 and Bcl-xL limit the activity of chemotherapeutic agents [17, 20]. The nuclear translocation of MCL1 further contributes to this chemoresistance phenotype. Therapeutic agents that target the anti-apoptotic Bcl-2 family members are currently in development for oncology indications in combination with site of care chemotherapeutics [6, 15, 35]. Since Bcl-2 expression is limited in CRC tumor samples (Fig. 5B), we subsequently sought to understand the impact of nuclear MCL1 on the activity of the Bcl-xL inhibitor A-1331852. Consistent with other reports [17], MCL1 knockout cells are sensitive to A-1331852 which is associated with apoptotic cell death as illustrated by the loss of viability and caspase-3 cleavage (Fig. 6A, B). Contrarily, MCL1 knockout cells expressing the loop domain mutant MCL1-∆198–207, in which MCL1 nuclear localization is blocked (Fig. 3A), were resistant to A-1331852 and were even resistant to doxorubicin/A-1331852 double treatment while cells expressing wild type MCL1 were resistant to A-1331852 or doxorubicin treatments (Fig. 6A, B). Although, expression of full length or loop dominant mutant of MCL1 inhibited A-1331852-medited apoptosis, this was overcome by co-treatment with the MCL1 small molecule inhibitor A-1210477. We showed that MCL1 nuclear translocation induced by doxorubicin makes cells more sensitive to cell death mediated by Bcl-xL inhibitor treatments. Mutations in the loop domain can block MCL1 nuclear translocation, therefore maintaining MCL1’s mitochondrial localization and anti-apoptotic function. Our data indicate that MCL1 and Bcl-xL cooperate at the mitochondria level to promote cancer cell survival, and MCL1 nuclear localization reconstitutes sensitivity to Bcl-xL inhibitors, phenocopying functional inhibition, or genetic ablation of *MCL1* as is illustrated in our proposed model (Fig. S3B).

To test the relevance of these observations in vivo, we used NSG mice bearing HCT116 p53^−/−^ tumors with wild type or CRISPR *ENO1*, in which MCL1 nuclear translocation in inhibited. Next, NSG mice were treated with Bcl-xL inhibitor A-1331852 with/without doxorubicin to induce MCL1 nuclear translocation. Our in vivo data revealed that only double treatment could inhibit HCT116 p53^−/−^ cell growth (Fig. 6C) and lengthen the survival time of mice with injected tumor cells (Fig. 6D). Conversely, growth of *ENO1*-deficient HCT-116 p53^−/−^ tumors was inhibited by doxorubicin but not A-1331852; adding A-1331852 to doxorubicin did not further inhibit tumor growth compared to doxorubicin alone (Fig. 6E). IHC of tumors harvested from mice confirmed that doxorubicin-induced MCL1 nuclear translocation was blocked in *ENO1* knockout cells (Fig. S1E). Consistently, we demonstrated that doxorubicin treatment was effective at abrogating tumor growth, but chemotherapy-induced DNA damage as illustrated *via* γH2Ax staining did not elevate caspase-3 (apoptosis) level in *ENO1*-deficient HCT116 p53^−/−^ tumors. On the contrary, apoptosis induced by A-1331852 in combination with doxorubicin was markedly abrogated in *ENO1* knock-out tumors (Figs. 6F, G and S1F, G). Collectively these data imply that tumor resistance to doxorubicin mediated by MCL1 nuclear translocation can be overcome by treatment with small molecule inhibitors of Bcl-xL, A-1331852, but this mechanism is dependent upon ENO1.

## Discussion

In this study, we illustrate that chemotherapy drives MCL1 from its canonical mitochondrial localization to the nucleus imbuing chemoresistance. We show for the first time that MCL1 accumulates in the nucleus in response to chemotherapy through an importin-independent, but ENO1/CaM-dependent pathway. Although the accumulation of nuclear MCL1 drives chemoresistance, this relocation process sensitizes cells to apoptosis driven by selective inhibition of Bcl-xL, advocating for combining Bcl-xL selective inhibitors with DNA damaging agents in patients with CRC.

The ability of MCL1 to inhibit the induction of apoptosis is well defined, facilitating tumor initiation and maintenance and driving chemoresistance [29, 36]. However, the data reported herein bolsters recent reports identifying MCL1’s non-apoptotic functions [24, 26, 37]. Our data indicate that MCL1 resists the cellular effects of chemotherapy not only through inhibiting apoptosis at the mitochondrial level, but also through nuclear accumulation [30, 31]. By utilizing cell lines expressing mutants of MCL1, we demonstrate that chemoresistance mediated by the nuclear translocation of MCL1 essentially requires the central loop domain; the BH3 domain being completely dispensable. Others have shown that MCL1 will translocate to the nucleus when residues within the C-terminus are altered or deleted [37–39]. Our data are the first to highlight a role for the loop domain of MCL1 to be important for tumor cell-survival and resistance to chemotherapy.

Although the vast majority of studies focus upon protein interactions with MCL1’s BH3 domain, we have identified a number of proteins that bind with MCL1’s loop domain including those known to have nuclear, transcriptional, and post-transcriptional activities. One of these proteins, CaM has an established role in nuclear transport through a Ca2^+^ dependent mechanism [40]. In turn, calcium release is a mechanism by which chemotherapies such as doxorubicin induce cell damage in cancer cells [41]. However, in silico evaluation showed that MCL1 does not have a CaM binding sequence. This contrasts with ENO1, which we demonstrate through immunoprecipitation experiments, binds to CaM and the loop domain of MCL1. Importantly, deletion of *ENO1* in HCT116 p53^−/−^ cells prevents doxorubicin-induced MCL1 nuclear translocation. The identification of ENO1 as a bridge between MCL1 and CaM adds significantly to the discovery of how MCL1 translocates to the nucleus. Although ENO1 traditionally is considered a glycolytic enzyme, it has other functions including a role in the metastatic potential of cancer [42, 43]. In fact, upregulation of ENO1 is associated with worse prognosis in many cancer types [44–46], which may in part be mediated by chemotherapy-induced nuclear translocation of MCL1 to drive chemoresistance, possibly through the regulation of transcription [47]. MCL1 could play a critical role in modulating and regulating the transcriptional activity of its binding partners, leading to mechanistic alteration of functionality. Our results collectively suggest that targeting ENO1 could become a novel intervention for preventing the chemoresistance of tumors.

CRC cell lines possess *Bcl-2L1* amplifications that associate with sensitivity to cell death mediated by selective Bcl-xL inhibitors [17]. Consistent with this, IHC analysis of primary human CRC patient samples confirm high Bcl-xL expression in CRC tumors harvested from both primary and metastatic sites as well as the near absence of Bcl-2 expression, which could serve as a resistance factor. However, there was also a high probability that MCL1 was co-expressed to medium/high levels in CRC tumor samples with medium/high expression of Bcl-xL and this was more common in stages III−IV than stages I−II. We and others have showed the association of MCL1 and Bcl-xL expression with tumor progression and survival in solid tumors including CRC [30, 48–52]. To our knowledge, there is no report on the association of the combination of MCL1 and Bcl-xL expression. Although, single protein Bcl-xL and MCL1 was able to stratify CRC outcome, a combination of Bcl-2, Bcl-xL, MCL1, Bax, and Bak was a better predictor of clinical outcomes, with Bcl-xL and MCL1 being the key dominating proteins [53]. Pre-clinical data emphasizes that Bcl-xL and MCL1 function as resistant factors to small molecule inhibitors that respectively target these two anti-apoptotic proteins [15]. Thus, genetic ablation of *MCL1* makes cancer cells sensitive to the targeting of non-MCL1 Bcl-2 family members such as Bcl-xL in CRC [17]. Our data indicate for the first time that MCL1 nuclear localization imbues sensitivity to Bcl-xL inhibitor A-1331852 in vitro and in vivo, phenocopying the impact of genetic ablation of *MCL1* on the activity of A-1331852 in CRC cell lines. Alternatively, preventing MCL1 from localizing to the nucleus through the expression of MCL1^Δ198–207^ or *ENO1* deletion maintains Bcl-xL inhibitor resistance, which can then be overcome with the MCL1 small molecule inhibitor A-1210477. Understanding the ability of different chemotherapeutics to drive the nuclear translocation of MCL1 could be key to developing combination strategies with Bcl-xL inhibitors, overcoming chemoresistance, and improving CRC patient outcome.

In summary, we demonstrated that nuclear MCL1 plays a role in mediating chemoresistance in colorectal tumors with aberrant or loss of p53 function. Importantly, we discovered a key mechanism of chemoresistance via induction of MCL1 nuclear localization through a novel binding partner, ENO1. A yet unexplored functional role of nuclear MCL1 could be to act as a chaperon of pro-oncogenic proteins into the nucleus that promotes tumor progression and chemoresistance. MCL1 seems to also function as a transcriptional activity regulator of its binding partners. There could be a bi-directional relationship between MCL1 and ENO1, where ENO1 shown to be involved in chemoresistance including nuclear translocation to inhibit transcription of proto-oncogene c-myc [54]. While nuclear MCL1 contributes to chemoresistance beyond its established anti-apoptotic function, it simultaneously increases the responsibility of inhibiting apoptosis to Bcl-xL, revealing a synthetic lethality with DNA damaging agents in the absence of functional p53. Since we demonstrate that MCL1 and Bcl-xL are frequently co-expressed at high levels in late-stage CRC patients, combining DNA damaging agents with selective Bcl-xL inhibitors may represent a tractable therapeutic strategy for treating this devastating disease.

## Supplementary information

Supplementary Figure 1

Supplementary Figure 2

Supplementary Figure 3

Supplementary Figure Legend

## Data Availability

All data supporting the findings of this study are available within this published article and supplementary information.
