# Supplementary figures and images for: MCL1 nuclear translocation induces chemoresistance in colorectal carcinoma
Source: Cell Death Dis. 2022 Jan 18;13(1):63. doi: 10.1038/s41419-021-04334-y (PMC8766550; doi:10.1038/s41419-021-04334-y)

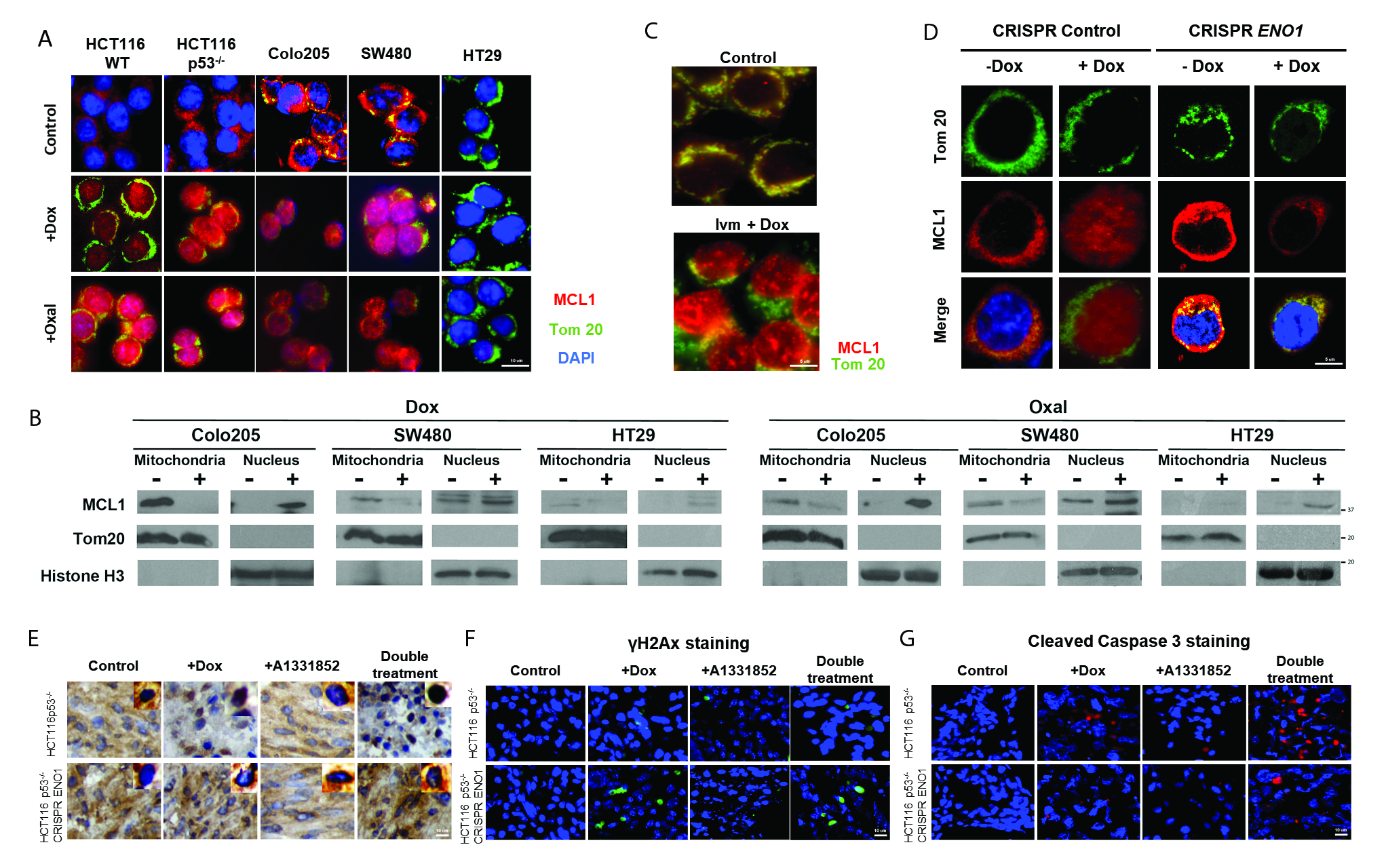

Supplement: Supplementary file 1 — Supplementary Figure 1 [file 41419_2021_4334_MOESM1_ESM.tif]

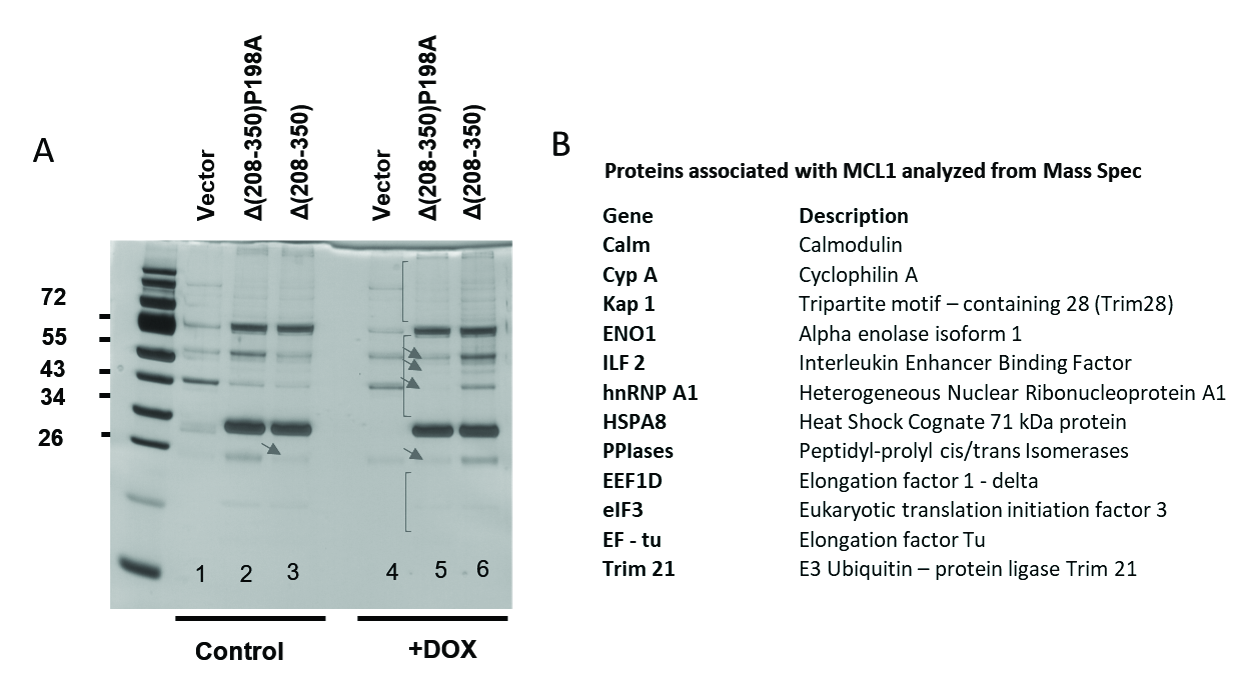

Supplement: Supplementary file 2 — Supplementary Figure 2 [file 41419_2021_4334_MOESM2_ESM.tif]

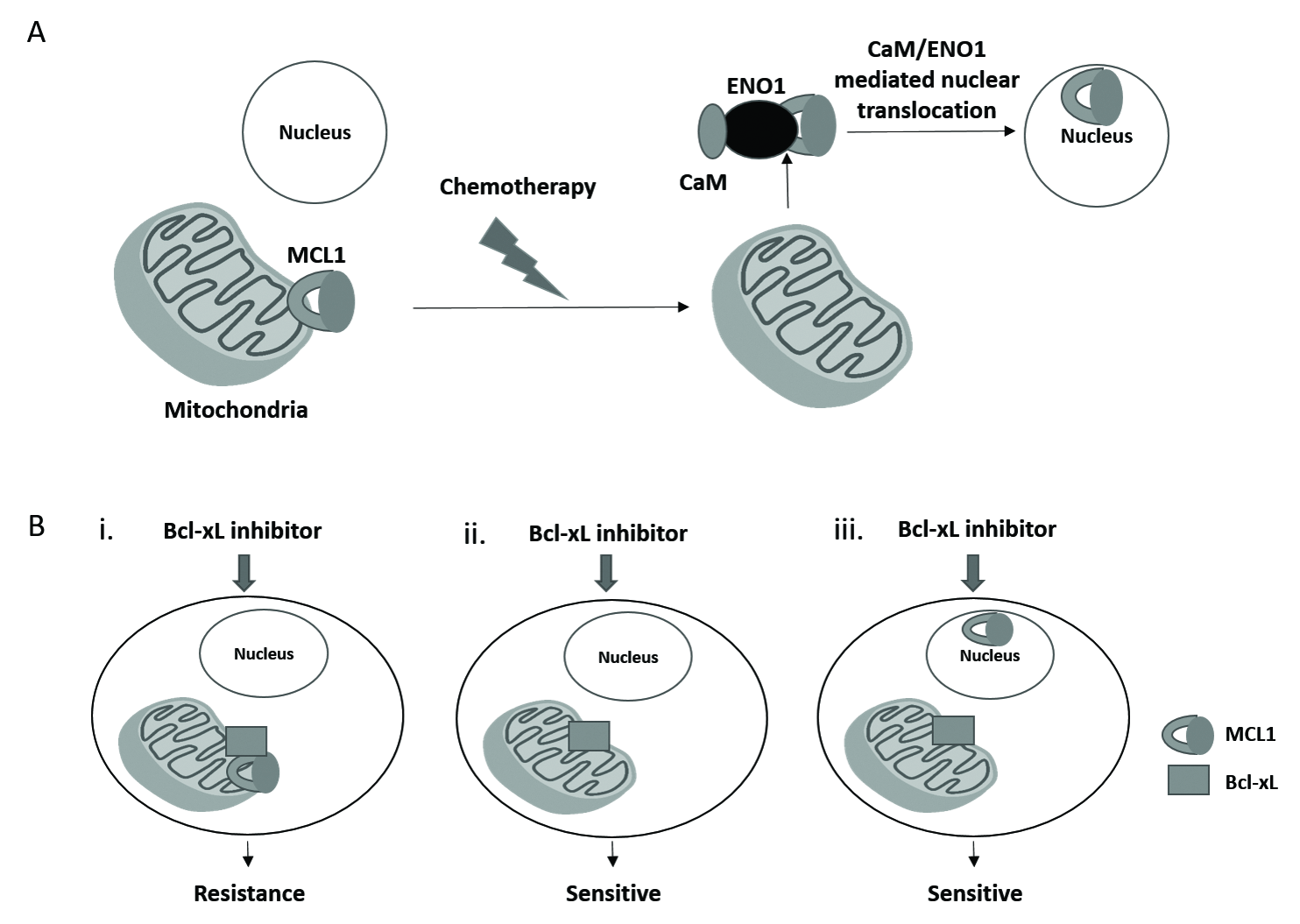

Supplement: Supplementary file 3 — Supplementary Figure 3 [file 41419_2021_4334_MOESM3_ESM.tif]
