# Supplementary material for: MCL1 nuclear translocation induces chemoresistance in colorectal carcinoma
Source: Cell Death Dis. 2022 Jan 18;13(1):63. doi: 10.1038/s41419-021-04334-y (PMC8766550; doi:10.1038/s41419-021-04334-y)
Supplement: Supplementary file 4 — Supplementary Figure Legend [file 41419_2021_4334_MOESM4_ESM.docx]

**Supplementary Figure Legends**

Figure S1

**Representation of microscopy images of cultured cells and tumor samples.** **A.** Representation of microscopy images with lower magnification of CRC cell lines: HCT116 wildtype (WT), HCT116 p53^-/-^, Colo205, SW480 and HT29 treated with Dox (100ng/ml) (middle panel) and Oxal (10μM) and no drug treatment control (top panel). Cell were stained with anti-Tom20 (Green) and anti-MCL1 (Red) antibodies. Nucleus were stained with DAPI (Blue). **B.** Mitochondria extraction and nuclear extraction were isolated from Colo205, SW480 and HT29 cells treated with doxorubicin (100ng/ml) or oxaliplatin (10uM). No drug treatment was used as control. Western blot was used to detect protein level of MCL1. Tom 20 were used as indicator of mitochondria and Histone H3 were used as indicator of nucleus. **C.** Nuclear translocation of MCL1 under CIS condition. HCT116 p53^-/-^ cells were treated with the importin inhibitor ivermectin (Ivm, 10 µM) for two hours followed by treatment with 100 nM doxorubicin for 24 hrs. Cells were then fixed and stained for MCL1 (red) and Tom20 (green). No drug treatment was used as control. **D.** Representation of confocal microscopy images of HCT116 p53^-/-^ with CRISPR *ENO1* and control cells treated with Dox (100ng/ml) (right panel) and no drug treatment control (left panel). Cell were stained with anti-Tom20 (Green) and anti-MCL1 (Red) antibodies. Nucleus were stained with DAPI (Blue). **E.** Representative xenograft tumor tissue sections from 6C were analyzed by staining with MCL1, with H&E background staining. Staining of MCL1 protein in single cells were shown in enlarged picture on the top of right side within each represent data. **F-G.** Representative xenograft tumor tissue sections from 6C and 6E were analyzed by staining with γH2AX nuclear bodies (F) and cleaved caspase 3 (G).

Figure S2.

**Protein identified to interact with the loop domain of MCL1 from mass spectrometry.** **A.** Silver staining to show the proteins pulled down by anti-Flag antibody from *MCL1* CRISPR HCT116 p53^-/-^ cells transfected with Flag tagged MCL1 protein with wild type or mutant loop domain. Arrows show the protein bands cannot be pull down by loop domain mutant Mcl-1 protein, comparing to wild type MCL1 protein. **B.** list of proteins identified from mass-spec assay that interact with wild type loop domain of MCL1.

Figure S3.

**A.** Modal of ENO1 mediated Calmodulin dependent MCL1 nuclear translocation**.** MCL1 proteins, release from mitochondria upon chemotherapy treatment, and bind to calmodulin via ENO1 proteins, which promote its nuclear translocation. **B.** (i) Modal of MCL1 and Bcl-xL dependent resistance to Bcl-xL inhibitor A-1331852 treatment in HCT116 p53^-/-^ Cells. (ii) MCL1 knockout or (iii) doxorubicin induced MCL1 nuclear translocation made HCT116 p53^-/-^ cells sensitive to Bcl-xL inhibitor A-1331852 treatment.

We have some minor changes in the supplementary figure legend:

- Fig. S1E : change from "... tissue sections from 7A were..." to "... tissue sections from 6C were..."
- Fig. S1F-G : change from "... tissue sections from 7A and 7C were...." to "...tissue sections from 6C and 6E were..."
